# Bibliographical Notices

**Published:** 1851-10

**Authors:** 


### BIBLIOGRAPHICAL NOTICES.

*A Practical Treatise on the Diseases and Injuries of the Urinary Bladder, the Prostate Gland and Urethra.* By S. D. Gross, M. D., Professor of Surgery in the University of Louisville, &c. pp. 726.

The late Dr. Parrish used to remark, that the "Cream of Surgery" was contained in the pelvis; and those who had the benefit of his wise instructions, will ever remember how his intelligent and benevolent face brightened up when he reached that part of his Surgical course relating to Hernia and Diseases of the Urinary Organs. With what interest he invested the subject, as he carried his hearers along with him, through the various difficulties which would meet them in the management of this class of cases, and as he recounted the triumphs of surgery in overcoming them.

There is, perhaps, no branch of Surgery, where the skill of the surgeon is more severely tested, and which should be more closely studied by every one who presumes to minister to the relief of surgical maladies.

Hence it is, that we hail the appearance of Dr. Gross' work with peculiar pleasure, as containing a thorough and systematic exposition of this class of diseases, arranged with great care and judgment, and written in a style of clearness and precision which must commend it to general acceptance.

The first five chapters are occupied with a lucid anatomical description of the urinary apparatus illustrated by a series of well executed wood cuts, with an account of the properties of urine, in its healthy and diseased conditions.

The diseases of the bladder are next treated of, including acute cystitis, fibrinous exudation of the bladder, suppuration and abscess, gangrene and ulceration.

The infrequency of acute inflammation of the bladder, is thus remarked upon by Dr. Gross:

"In the course of an extensive practice during the last twenty years, comparatively few cases of this complaint have fallen under my observation, nor has this organ, in the numerous dissections which I have made within that period, exhibited, except in a few instances, evidences of this affection. Dr. Louis, of Paris, examined the mucous membrane of the bladder in five hundred subjects, dead of various diseases, without discovering any serious lesion in any of them. In six there was simple redness or injection of the vessels, but no change of structure; in a few only, was there any softening and organic derangement. Similar testimony in regard to the infrequency of acute inflammation of the bladder is borne, by Brodie, Hope, Begin, Coulson, and other writers on the diseases of the urinary apparatus."

As an idiopathic affection, there can be no question of the great rarity of cystitis, while as a symptom of other diseases of the urinary organs, it often occurs.

Thus we see all the evidences of high irritation and inflammation of the mucous coat, in the mechanical obstructions of the urethra which impede the passage of urine, in the extension of gonorrhœal inflammation from the urethra to the bladder, in strangury, &c., requiring treatment adapted to the circumstances of each case. The remedies recommended for cystitis in its several forms and stages are fully detailed, and appear to be well chosen and judicious. The following remarks on the use of anodyne injections in this affection, are applicable to a large class of cases, not included under this head, and are well worthy of attention.

"Of all the local remedies none hold a higher rank in the treatment of this affection than anodynes, administered by the rectum, either in the form of *injections* or in that of suppositories. They not only allay pain and spasm, but they quiet the bladder, and render it more able to bear the presence of the urine, a desire to pass which is the principal cause of the patient's suffering. The best form of injection is from half a drachm to a drachm and a half of laudanum to two ounces of tepid water, thrown up with a good pewter syringe, with a long nozzle, which is far preferable to all the patent contrivances of the kind of which I have any knowledge. The bowel should be previously cleared out with a purgative, or an enema, and care should be taken not to force the fluid against the anterior wall of the rectum. The quantity of laudanum here specified is a dose for an adult; for a younger subject, or a person enfeebled by age and disease, a smaller quantity will suffice. The repetition of the medicine must be regulated by circumstances; if it pass off soon after it is administered, it should be immediately renewed; and the same rule should be enforced, if it is retained, if it does not answer the desired end in two or three hours. Where laudanum is inadmissible, on account of idiosyncrasy, black drop, or morphia, may be employed as a substitute."

The other sections of this chapter are occupied with the consideration of fibrinous exudation of the bladder, suppuration and abscess, gangrene and ulceration. But these morbid conditions are so rare, as scarcely to demand notice, except in connection with other diseases of the organ of which they form one of the stages.

Under the head of "Chronic lesions of the bladder," we have a chapter on Catarrh of the bladder, or Cystorrhœa. Under this term Dr. Gross includes all those affections of the mucous coat attended by an inordinate mucous secretion, whether arising from stricture of the urethra, the presence of calculus, hypertrophy and enlargement of the prostate gland, &c.

Regarded in this light, cystorrhœa is nothing more than a symptom of a more serious malady, and would scarcely seem to be entitled to consideration as a distinct disease.

Thus Dr. Gross remarks:

"Cystorrhœa is always dependent, directly, or indirectly, upon some obstacle to the evacuation of the urine, or upon a diseased condition of the bladder itself. Hence the most common exciting causes are stricture of the urethra, the presence of a calculus, hypertrophy, and enlargement of the prostate gland. In fact, there are few protracted cases of this kind in which this affection is not witnessed to a greater or less extent, or of which it does not constitute in the end a prominent symptom. Nearly all the very worst forms of vesical catarrh I have ever seen have been of this description. Paralysis of the bladder, whether produced by over-distension of the organ by urine, or injury or disease of the spine, frequently gives rise to this state. The muscular fibres having lost their expulsive power, the water is never completely evacuated at any one time even when the catheter is used, but a portion remains in the bottom of the bladder, where it is speedily decomposed, and thus acts as an irritant to the lining membrane, followed by an inordinate secretion of mucus. Cystorrhœa is a constant attendant upon sacculation, ulceration, hypertrophy, and carcinoma of the bladder."

Now we do not consider the increased and altered mucous secretion, dependent upon mechanical obstruction to the passage of urine, or to the presence of stone, as constituting catarrh of the bladder, strictly speaking, nor can we admit the propriety of so considering it.

In the treatment of cystorrhœa Dr. Gross fully recognizes the distinction between the idiopathic affection, and that which is merely symptomatic. In the latter form, the nature of the exciting cause must be ascertained, while the former must be reached by antiphlogistics, balsams, terebinthinates, &c. The merits of the several remedies recommended by authors in this form of disease, are briefly discussed, and much valuable practical information conveyed to the readar. Dr. Gross himself places a high estimate upon antiphlogistics.

"It would be useless to repeat here what has been already said, in other portions of this treatise, respecting the employment of antiphlogistics. The propriety of these measures is self-evident. They are imperatively demanded in all cases attended with violent pain and frequent micturition, even when there is no marked constitutional disturbance. The abstraction, under such circumstances, of fifteen or twenty ounces of blood from the arm, will often do more good in breaking up the disease than any other remedies that we possess. Where the lancet is inadmissible, twenty or thirty leeches may be applied to the perinæum and inside of the thighs, or to the lower part of the hypogastric region."

We must dissent from this indiscriminate recommendation of the lancet in a disease almost confined to aged persons, and often connected with an enfeebled state of the system. The abstraction of fifteen or twenty ounces of blood from an old man who has "violent pain and frequent micturition, even when there is no marked constitutional disturbance," would appear to us injudicious, especially as we have often seen these symptoms promptly yield to emollients, anodynes, warm bathing, &c.

A peculiar form of hypertrophy chiefly affecting the neck of the bladder, and generally found in old subjects, is next described under the name of "bar like ridge of the neck of the bladder," with a wood cut, affording a beautiful illustration of this disease, from a specimen in the cabinet of Dr. Sabine of New York. This affection is generally connected with enlarged prostate, and like it, does not admit of cure.

The chapter devoted to nervous affections of the bladder, is one of the most interesting and instructive portions of the work. Irritability and neuralgia of the bladder are accurately described, and the various points of diagnosis between them and organic lesions of the organ are pointed out. Dr. Gross' remarks upon the causes of this irritability, and upon the means of cure adapted to particular cases, convey a large amount of valuable instruction upon a subject which has received but little attention from medical writers; we regret that our limits will not admit of making extracts, but we must forbear.

Paralysis of the bladder, from external violence, from over disension, from want of power in the general system, constitutional irritation after operations, from low fevers, &c., are treated of under appropriate heads. The following directions on the use of the catheter in these cases, are sound and judicious, and worthy to be borne in constant rememberance by the practitioner.

"Two important indications are presented in every case of this disease; first, to draw off the urine, and secondly, to restore the tone of the muscular fibres of the affected organ. To fulfil the first, all that is necessary is to use the *catheter*. This should be done at stated intervals, to prevent undue accumulation, and to compel the viscus to return, as it were, to its original habits. Carefully persevered in, this practice is frequently of itself sufficient, in a short time, to cure the malady. In confirmed cases,the instrument should be employed once about every four hours, especially if there be much renal secretion; in opposite states, on the contrary, three or four times a day will be often enough. I generally prefer introducing the catheter every time it is necessary to draw off the urine to letting it remain in the bladder permanently; and as there is seldom any difficulty in doing this, the patient usually soon learns to perform the operation himself. Sometimes, however, the improvement is more rapid and decided when the catheter is constantly retained, and the water permitted to flow off every hour or two. I have found this practice particularly useful in cases of paralysis, attended with pain and spasm of the neck of the bladder, and a frequent desire to urinate. When the accumulation is very great, and has continued for several days, it is a good rule not to evacuate all the water at once, for fear of inducing severe depression from the sudden removal of the stimulus of distension. I have seen several cases in which I am satisfied the patients lost their lives from inattention to this precaution. My own practice, under such circumstances, is not only to allow a small quantity of urine to remain, but to support the weakened organ by swathing the abdomen, precisely as after parturition, and tapping in ascites. When the catheter is permanently left in the bladder, it should be confined in the usual manner, and cleaned every other day; otherwise it will be certain to become encrusted with inspissated mucus, if not with earthy matter, and thus produce an injurious impression upon the affected organ.

Much harm is often done in this disease by the protracted employment of the catheter. The proper plan is always to discontinue it as soon as it is discovered that the organ has regained its expulsive power. The patient should be requested from time to time to try to evacuate the bladder by his own efforts, and if he is not able to effect the object completely, he should be assisted with the catheter; for the rule is, in all cases, to draw off every particle of water at least twice in the twenty-four hours. By employing the instrument too long, the organ becomes habituated to its use, and a much longer time will necessarily elapse before a cure takes place."

Chapters VI and VII are ocupied with a description of schirrous, encephaloid and tubercular diseases of the bladder,with other non-malignant growths; all of which rarely occur in practice, but which must have a place in a systematic treatise on the surgery of this part of the body.

Chapter VIII is a curious essay on worms in the bladder, a few cases of which have been reported by medical writers. Serous cysts and hydatids, fœtal remains in the bladder, hair and air in the bladder, are each considered, though they possess but little practical interest.

An interesting chapter is devoted to vesical hemorrhage, both idiopathic and traumatic. Considering the frequency of this accident from unsuccessful and awkward attempts to introduce the catheter, or from well directed efforts in diseased conditions of the neck of the bladder and prostate gland, we were surprised to find so little notice of it in this connection. There are fewer cases presenting greater difficulties, in the introduction of the catheter, and the evacuation of the bladder, than these, and it would seem desirable in a work like the present, to have laid down the principles upon which they should be treated more in detail.

The following extract conveys the author's views on this point:

"In general, the blood which is poured out in vesical hemorrhageis dissolved by the urine, and thrown off by the natural channel. In some instances, however, especially when the secretion of urine is deficient, or the quantity of blood disproportionably large, the accumulated fluid coagulates and distends the bladder, which forms a hard, firm tumor above the pubes, and leads to complete retention of urine, attended by the most urgent and distressing symptoms. To free the organ of its contents, under such circumstances, is often no easy task. The more simple means are of course resorted to first. With a silver catheter, introduced along the urethra, an attempt is made to break up the coagulated mass, and then to dissolve the pieces by the injection of tepid water and acetic acid, in the proportion of one ounce of the latter to five ounces of the former. Vinegar is a powerful solvent of blood, and is far better than water alone. The injections should be conducted with great care, and should be repeated two or three times in the twenty-four hours. Some of the smaller coagula may sometimes be removed by a syringe applied to the catheter, though such a procedure is, in general, quite inefficient."

Retention of urine, catheterism, and puncture of the bladder, are treated of in chapter XIV. Dr. Gross prefers the silver to the gum elastic catheter in all cases of retention from hypertrophy of the prostate gland. On this point he remarks:

"The treatment is by the catheter; and one of silver is far preferable to one of gum elastic. It must be large in the curve, and at least two inches longer than in ordinary cases, otherwise it will fail to reach the distended reservoir. The instrument passes on without difficulty until it comes in contact with the enlarged gland, when its progress is arrested. Instead of forcing it onward, the surgeon introduces the left index finger, well oiled, into the rectum, and placing it against the instrument, he guides its beak into the bladder, by pushing it gently towards one side or upwards towards the pubes, at the same time that he urges the handle on with the right hand. By this manœuvre the obstacle is usually overcome without much trouble, however great the enlargement of the prostate. To empty the bladder completely it is necessary, as the point of the catheter cannot reach the cavity behind the gland, to raise the patient's hips, so as to force the urine out of its hiding-place."

Although there can be no question of the advantages of the silver catheter in cases of difficult obstruction, yet it must be confessed that there are a multitude of cases where the gum elastic instrument is preferable. As for instance: in that large class where the daily use of the instrument for a lengthened period becomes necessary in ordinary cases of retention without obstruction, or in those in which obstruction has been overcome by the previous use of a silver instrument.

We presume Dr. Gross would favor the use of the gum elastic catheter in these cases, and yet we find no mention of it in his remarks on the subject; thus conveying the impression that the silver catheter should be mainly relied on in practice, a recommendation which we consider far too indiscriminate.

Under the head of "puncture of the bladder," Dr. Gross discusses the circumstances under which it may become necessary to reach the bladder, either by the rectum, perineum, or above the pubis; and each of these operations is described. The necessity for their performance, however, is extremely rare, and some surgeons have even doubted the propriety of it under any circumstances.

In the chapter on Incontinence of Urine, we find the following practical hints on the management of this troublesome affection occurring in children:

"In that variety of the affection which is met with in boys and girls, the cure may be greatly expedited by proper attention to the diet, which should always be bland and unirritant. Late suppers are avoided, and the patient must abstain entirely from drinks for several hours before going to bed. During the night he is to be waked two or three times for the purpose of emptying his bladder, and this practice is to be persisted in for weeks, and even months, until the disagreeable habit is broken up. During all this time, as well as, indeed, for a long period afterwards, the child should lie upon his side, to prevent the urine from coming in contact with, and irritating the neck of the bladder. The internal remedies from which I have derived most benefit in the treatment of this affection, are strychnine and cantharides, given three times a day, in the proportion of the twelfth or sixteenth of a grain of the former to the eighth or tenth of a grain of the latter, according to the age of the subject. A minute portion of opium forms a valuable addition: and, in atonic cases, I often combine with these articles some of the preparations of iron. When the strychnine disagrees, or fails to answer the purpose, we may substitute the extract of nux vomica. In either case, it is important to watch the effects of the remedy. I have great confidence in the use of cantharides in this affection, and have known it to afford relief when everything else seemed to prove unavailing. I prefer the powder to the tincture, and occasionally continue the exhibition of it until slight strangury is induced."

Our author does not seem to have been aware of the valuable paper of the late Dr. Otto, of Philadelphia, on this subject, published in the *North American Medical and Surgical Journal*, in 1830. The treatment introduced by Dr. Otto, and since successfully practised in this city, consists of the free use of uva ursi tea, and the muriated tincture of iron, with cold dash, blister to the sacrum, &c.

Chapter XVII enters into an elaborate examination of the subject of urinary deposits, their origin, chemical constitution, and the modes of medical treatment adapted to the several varieties. And chapter XVIII treats at large of "Stone in the Bladder, its nature and causes, symptoms, and the different methods of operating for its relief." This chapter occupies 173 pages, and is a full exposition of the subject, bringing before the reader the latest improvements in the several operations for stone, and comparing with judgment their relative merits.

Our space will not permit us to follow the author through this interesting portion of his work, but we can recommend its careful perusal and study, especially to the younger members of the profession who wish to be thoroughly acquainted with this important branch of surgery.

The Diseases and Injuries of the Prostate Gland form Part II. of the volume.

These affections are considered in their proper order, and are illustrated by a series of handsome wood-cuts. We regret that we must also pass over the many interesting points embraced in this part of the work, for although our author has not been able to bring forward anything new in the treatment of these diseases, yet he has presented the existing state of knowledge on the subject, in a clear and perspicuous manner.

Part III treats of the "Diseases and Injuries of the Urethra," including rupture or laceration, stricture, tumors of the urethra, neuralgia of the urethra, foreign bodies in the urethra, infiltration of urine, fistula, false passages, &c. &c., covering 116 pages. A large amount of practical matter of great interest is here brought together, but we are prevented from even presenting a glimpse of it to our readers.

We can cordially recommend the volume of Dr. Gross both to the practitioner and student, as an excellent systematic treatise on a class of diseases both common and important. It is highly creditable to him, and no less so to our national medical literature, yet struggling for an existence, but destined in no distant day to form an important part in the great republic of medical letters.

---

*Southern Medical Reports.* Edited by E. D. Fenner, M. D., of New Orleans. Vol. I. 1851.

We regret that the late period at which we received this interesting volume, has prevented our giving it that extended notice which its merits demand. At some future time, not far distant we trust, we will lay before our readers a digest of its contents. In the mean time we bespeak for it the earnest attention of the profession in the North as well as in the South, as containing matter that will amply repay persual.

---

*Special Anatomy and Histology.* By William E. Horner, M. D., Professor of Anatomy in the University of Pennsylvania, Senior Surgeon to the St. Joseph's Hospital, &c. *Eighth edition, Illustrated with anatomical figures. In two volumes.* Philada. Blanchard & Lea. 1851.

A new edition of this popular and standard treatise on Anatomy having been called for, the author has incorporated into it such improvements as the advancing condition of the science required. The text has been, in many places, modified in accordance with existing views, and a number of new illustrations added to it. Upon a work so extensively known, and so generally acceptable, it would be useless to comment; it has already received the most convincing proof of its popularity and usefulness in its rapid sale. Having announced to our readers, therefore, this new issue, we commend it to all as one of the best books on the subject in the English language.

---

*The Laws of Health, in relation to Mind and Body.* By LIONEL JOHN BEALE, M. R. C. S. Philadelphia, Lea & Blanchard, 1851. pp. 295.

This is a sensible and agreeably written work on a somewhat hackneyed subject. We feel, however, altogether disposed to admit the force of the author's plea, that, "although there are many works on health addressed to the public, yet it cannot be said that their influence has been sufficient to supersede the necessity of another, while such manifold evidence continues of the desolations which afflict mankind from ignorance of the laws of health."

Books like this are certainly of the best tendency, and offer the most effectual antidote to the spread of quackery and charlatanism. We can recommend Mr. Beale's little volume as especially deserving of professional countenance and support. It contains a very clear resumé of the leading facts of physiology, with an excellent manual of dietetic and hygienic recommendations; and, although, perhaps, a little overloaded with metaphysical discussion, presents much that is truly admirable in the connection between intellectual and moral improvement and the maintenance of health.

We ask the attention of our readers to the following remarks on empiricism and false theories of medicine:

"Among the diseases to which the human frame is liable, of course many are incurable; and it is on the fears of persons thus afflicted that the inventors of novel modes of treatment, the proprietors of nostrums, and pretenders of all sorts, make their harvest. The art of medicine is partly empirical; many of the remedies we use have been discovered to be so by accident or experience: hence the opinion, that there may be secret modes of cure, for particular diseases, in possession of persons who have never studied medicine. Upon this possibility, there has ever been a field for the owners of nostrums, the curers of disease by miracles, the projectors of new methods of treatment, as Homœopathy and Mesmerism, or the universal application of an old one, as Hydropathy.

As long as the world lasts there will be nostrums; every family remedy is one; and who can say that an old lady, who may have put together several simples, and formed a useful remedy for a cough, shall be prevented by law from giving or selling her medicine? Medical reformers argue, that all medical treatment should be confined to those who have proved their qualifications by proper tests; but the public will never admit of such an attack on liberty: nor would it be just, if it could be accomplished, for some very useful medicines have been nostrums, as James's powder. Until we can make medicine an inductive science, we have no right to ask for the exclusive privilege of medical treatment. The true method of putting down pretenders in medicine, as in every thing else, is to enlighten the public mind—it is ignorance which affords patronage to secret remedies, miraculous cures, and quackeries of all sorts, both in and out of the domain of physic. When all medical practitioners shall cease to be pretenders to more knowledge than they really possess, then will the public cease to patronize quackery;—and a more complete education—intellectual, moral and professional—of all classes of medical practitioners, engendering higher views of their duties, will cause them to rank higher in the estimation of the public, and be productive of greater benefit, than any exclusive privileges which the Legislature could confer upon them."

---

*Dispensatory of the United States of America.* By George B. Wood, M. D., Professor of the Theory and Practice of Medicine in the University of Pennsylvania, &c. &c., and Franklin Bache, M. D., Professor of Chemistry in Jefferson Medical College of Philadelphia, &c. &c. *Ninth Edition, carefully revised.* Philadelphia: Lippincott, Grambo & Co., 1851. pp. 1456.

The ninth edition of the U. S. Dispensatory, just issued, has closely followed the publication of the last, which appeared only two years since. This has, however, been rendered necessary, not only by the great accumulation of valuable additions to the Materia Medica during the period in question, but also by the recent publication of the decennial editions of the United States and British Pharmacopœias. The new edition of the Dispensatory is enlarged between sixty and seventy pages beyond the limits of the last. It is, perhaps, superfluous for us to say that it fully keeps pace with the progress of the Materia Medica and

Pharmacy ; and, in view of the very great mass of new materials which the authors have had to deal with, they have indeed been particularly successful in performing the difficult task of selection and condensation. The "ninth edition" in every way maintains the reputation of the Dispensatory as a standard national work.

---

*The Geological Observer.* By Sir HENRY T. DE LA BÉCHE, C. B., F. R. S., &c., Director General of the Geological Survey of the United Kingdom. Philadelphia. Blanchard & Lea, 1851. 8vo. pp. 695.

We have great pleasure in bringing to the notice of our readers the very handsome reprint of Sir H. De La Béche's excellent work on Geology, with which we have been favored by the publishers, Messrs. Blanchard & Lea. It is one of the best elementary works on the subject, in the language.

---

*A New Sign Language for Deaf Mutes.* By ALBERT J. MYER, Buffalo, 1851.

This pamphlet is a *thesis* for the degree of Doctor in Medicine, presented before the medical department of the University of Buffalo, and published in accordance with a vote of the faculty. The author, who appears to have been formerly connected with a telegraph office, recommends the employment of Bain's alphabet as a new sign language (meaning of course a new alphabet) for deaf mutes. According to his suggestion, the finger is made to write upon a table, or "the hand of a companion or any portion of his body," it being understood "that whenever the finger rests upon the table, it is supposed to be drawing a line." Dots and lines are the symbols used in Bain's alphabet, and "to form a *dot*, the hand is brought in contact with the table or other object, and instantly returned to the standard position. To form a *line*, it is brought down similarly, retained for a longer period in contact, and in like manner returned to place."

This is undoubtedly a practicable alphabet for deaf mutes. But it is more complicated, more difficult to acquire, and more open to mistakes in practice, than the ordinary alphabet with the fingers, which has moreover the advantage of being arranged to resemble the letters of the printed and written alphabet.

---

*A Historical Sketch of Surgery, being an Introductory Lecture to a course on Surgery, in Geneva Medical College, N. Y.* By James Bryan, M. D.

This is a very pleasantly written sketch of the history of surgery, "from the revival of literature, to the end of the seventeenth century."

